# Implementation of Nurse Staffing Policy and Patient Experience Assessment: A National Cross‐Sectional Study

**DOI:** 10.1155/jonm/1655763

**Published:** 2026-08-23

**Authors:** Sung-Heui Bae, Ye-Eun Jin

**Affiliations:** ^1^ College of Nursing, Graduate Program in System Health Science and Engineering, Ewha Womans University, Seoul, South Korea, ewha.ac.kr; ^2^ Graduate School of Nursing, Ewha Womans University, Seoul, South Korea, ewha.ac.kr

**Keywords:** comprehensive nursing care service, hospital environment, hospital evaluation, nurse staffing, patient experience, patient satisfaction

## Abstract

**Background:**

South Korea’s comprehensive nursing care service (CNCS) was introduced to alleviate caregiver burden and improve patient care.

**Purpose:**

The purpose of this study was to examine the association between the CNCS policy and patient experiences in South Korean hospitals.

**Methods:**

This cross‐sectional study was based on a secondary data analysis of data from the 2021 3rd patient experience assessment of 322 hospitals in South Korea. Multivariate regression was used to assess the relationship of interest while controlling for hospital characteristics.

**Results:**

Hospitals with 50%–100% CNCS beds had higher patient experience scores in nursing care (*β* = 1.39, *p* < 0.01), medication and treatment (*β* = 1.34, *p* < 0.05), hospital environment (*β* = 3.68, *p* < 0.01), and overall evaluation (*β* = 2.60, *p* < 0.01).

**Conclusion:**

Hospitals with more CNCS beds and better nurse staffing levels were significantly associated with higher patient experience scores across multiple domains. This study reinforces the importance of the CNCS policy as a critical factor contributing to enhanced patient satisfaction.

**Implications for Nursing Management:**

Nurse managers and policymakers should expand the CNCS and ensure adequate nurse staffing, which are consistently associated with better patient experience across domains. Because hospital environment and nursing care showed the strongest associations, hospital administrators can use these domains to monitor CNCS effectiveness and guide resource allocation.

## 1. Introduction

South Korea and other Asian countries maintain a unique caregiving culture wherein informal caregivers, either family members or hired caregivers, are permitted to reside in hospitals to assist with daily patient care activities [1, 2]. Consequently, compared with Western healthcare systems, fewer nurses care for a larger patient population [1]. In 2021, South Korea had 8.9 licensed nurses per 1000 people, which was below the Organization for Economic Co‐operation and Development (OECD) average of 12.2 per 1000 [3]. Moreover, the ratio of practicing clinical nurses in South Korea stood at 4.6 per 1000 individuals, which was also notably below the OECD average of 7.9 per 1000 [3]. Although the number of licensed nurses in South Korea is relatively high, a much smaller proportion of them actively work in clinical settings. This gap suggests potential challenges within the healthcare system, such as nurse retention, working conditions, and the distribution and allocation of healthcare resources.

As countries rapidly become aging societies, the caregiving burden is becoming increasingly severe, with continued increases in private caregiving costs. Caregiver service fees increased by approximately 26.24% from 2020 to 2023 [4]. Furthermore, the caregiving expenses incurred by patients and their families rose from 3.6 trillion won in 2008 to over 8 trillion won in 2018. This figure is projected to exceed 10 trillion won annually by 2025 [2]. To address these challenges, the Korean government introduced the comprehensive nursing care service (CNCS), commonly referred to as the Integrated Nursing Care Service System. It prohibits caregivers from staying in hospitals, allowing only visitation, and requires that all patient care be provided by a team comprising nurses, nurse assistants, and other caregiving staff [5, 6]. Under Article 4–2 of the Medical Service Act, the CNCS system refers to inpatient services without the presence of caregivers or family members, as defined by the MOHW [6].

In July 2013, a pilot program was launched in 13 hospitals, marking the first implementation of “caregiver‐free hospitals,” wherein individual caregivers were no longer permitted [5, 7]. In 2014, this pilot program was renamed the Comprehensive Nursing Service, under which all inpatient care was provided by assigning suitable nursing staff to patients without the participation of personal caregivers [7]. This project significantly transformed the nursing service delivery system and caregiving tasks within South Korea’s hospital‐centered care framework [1]. The number of hospitals offering these services increased from 28 in 2014 to 107 in 2015 [7]. Following the Middle East Respiratory Syndrome outbreak, tertiary hospitals were allowed to participate in the program starting in April 2016. By 2019, the government had expanded the availability of comprehensive nursing services to a larger number of hospitals, extending beyond national and public institutions to include private hospitals [1, 7]. Under Article 4–2 of the Medical Service Act, participation in the CNCS is voluntary for most hospitals but mandatory for public hospitals [6]. As the CNCS can be implemented at the nursing unit level rather than hospital‐wide, the proportion of CNCS beds varies depending on the extent of implementation within each hospital [8].

Overall, the number of hospitals operating the CNCS grew to 562 hospitals with 50,201 beds by 2020 [9]. As of June 30, 2024, the number of hospitals offering the CNCS had expanded to 745, accounting for approximately 23.5% of all eligible inpatient hospitals in South Korea [8]. The MOHW announced the plan to alleviate the national caregiving cost burden following consultation with governments [10]. It aims to reduce the caregiving burden by expanding the number of patients utilizing “caregiver‐free wards” under the CNCS, with a target increase from the current 2.3 million patients annually to 4 million by 2027 [10].

Previous studies have explored the relationship between the CNCS and patient satisfaction and found that inpatients who received the CNCS reported significantly higher satisfaction levels than those cared for by family members [11]. In addition, more positive nurses’ attitudes were associated with greater patient satisfaction, which indicates that providing high‐quality CNCSs can effectively enhance satisfaction among hospitalized patients [11].

Some studies have also examined the impact of CNCS policies on patient experiences [1, 7]. In South Korea, nurse staffing grades (NSGs), based on the bed‐to‐nurse ratio at the hospital level, are commonly used to assess staffing levels, with lower grades indicating better staffing. Lee et al. [1] found higher patient satisfaction scores in CNCS wards than in those without such services, reflecting the policy’s effectiveness and significant improvements in nursing quality. Choi et al. [7] indicated that introducing CNCS beds positively impacted patient experiences, particularly within the hospital environment domain, and had a marginally positive effect on overall patient evaluation. Furthermore, in hospitals with NSGs of level 2 or below, a higher level of implementation of CNCS beds was associated with higher patient experience assessment (PXA) scores, indicating that NSGs serve as a significant moderating variable [7]. Song and Do [12] utilized data from the PXA and found a strong association between nurse staffing levels and patient experience across multiple domains. This study used item‐specific data from the PXA, enabling a detailed analysis of patient experiences reported in response to each survey question. In particular, the results showed that lower nurse staffing levels were consistently associated with less favorable patient outcomes [12].

Despite the importance of nurse staffing levels in shaping patient experiences, a significant gap remains in the empirical evidence from South Korea [7, 12]. Although studies from countries such as the United States and the United Kingdom have consistently highlighted the critical role of adequate nurse staffing in ensuring positive patient outcomes [13, 14], similar research within the South Korean context is limited [7, 12]. Studies in Korea have primarily focused on individual‐level analyses, such as patient satisfaction with the CNCS, and have often overlooked broader organizational factors that may influence patient experiences [1, 7, 11]. This research gap underscores the need for studies that not only examine the association between nurse staffing levels and patient experiences but also investigate how NSG variations and the bed allocation to CNCSs affect overall patient satisfaction in South Korean hospitals.

### 1.1. Aim

This study evaluated the association of the CNCSs with patients’ experiences in South Korean hospitals. Specifically, this study examined how the percentage of CNCS beds influences PXAs across various domains, including nursing care, medical care, treatment processes, hospital environment, patient rights protection, and overall patient evaluation. In addition, the study assessed NSGs as an associated staffing factor, providing insight into how staffing adequacy affects patient‐centered outcomes.

## 2. Methods

### 2.1. Study Design and Sample

A cross‐sectional study used data from the third PXA provided by HIRA in South Korea [15]. The PXA was first introduced in 2017 to foster patient‐centered healthcare services [16]. The results of the first and second assessments in August 2018 and July 2020, respectively, were publicly disclosed at the hospital level. The third assessment was conducted in 2021 through a nationwide telephone survey to collect data from all general and tertiary hospitals [16].

Patients who responded to the PXA were those aged 19 years and above who were hospitalized for at least one day at the hospitals and were discharged within 2 to 56 days prior to the survey [16]. For this study, 322 hospitals were included based on publicly available data from the third PXA, comprising 47 tertiary and 275 general hospitals [15].

### 2.2. Data Collection

Data were collected from the third PXA in 2021, available on HIRA’s website as of September 6, 2021 [16]. Other data for the independent and dependent variables, including the percentage of CNCS beds [17], NSGs [18], hospital type [19], public or private hospital status [19], the percentage of beds in advanced hospital rooms [19], the percentage of medical specialists [20], the total number of high‐cost medical equipment items [21], the total number of beds [19], and the hospital location [19], were obtained from the public data portal (Table 1).

**TABLE 1:** Variable lists and data sources for analysis.

Variable type	Variable	Download portal	Data source	Third PXA 2021	Link
Dependent variable	Patient experience assessment (PXA)	Health Insurance Review & Assessment Service (HIRA)	Health Insurance Review & Assessment Service (HIRA)	2021	https://www.hira.or.kr/ra/eval/getDiagEvlList.do?pgmid=HIRAA030004000100
Independent variable	Percentage of comprehensive nursing care service beds	Download from the Public Data Portal	National Health Insurance Service_Operational Status of Comprehensive Nursing Care Services	2021	https://www.data.go.kr/data/15104803/fileData.do
Mediator variable	Nurse staffing grades (NSG)	Download from the Public Data Portal	Health Insurance Review & Assessment Service (HIRA)_ Detailed information service for hospital nursing staffing grade information	2021	https://opendata.hira.or.kr/op/opc/selectOpenData.do
Control variable	Hospital type	Download from the Public Data Portal	Health Insurance Review & Assessment Service (HIRA)_Detailed information service for hospital_01_Facility information	2021	https://opendata.hira.or.kr/op/opc/selectOpenData.do
	Hospital ownership	Download from the Public Data Portal	Health Insurance Review & Assessment Service (HIRA)_Detailed information service for hospital_01_Facility information	2021	https://opendata.hira.or.kr/op/opc/selectOpenData.do
	Location	Download from the Public Data Portal	Health Insurance Review & Assessment Service (HIRA)_Detailed information service for hospital_01_Facility information	2021	https://opendata.hira.or.kr/op/opc/selectOpenData.do
	Total number of beds	Download from the Public Data Portal	Health Insurance Review & Assessment Service (HIRA)_Detailed information service for hospital_01_Facility information	2021	https://opendata.hira.or.kr/op/opc/selectOpenData.do
	Percentage of beds in advanced hospital rooms	Download from the Public Data Portal	Health Insurance Review & Assessment Service (HIRA)_ Detailed information service for hospital_01_Facility information	2021	https://opendata.hira.or.kr/op/opc/selectOpenData.do
	Percentage of medical specialists	Download from the Public Data Portal	Health Insurance Review & Assessment Service (HIRA)_Hospital Information Service	2021	https://opendata.hira.or.kr/op/opc/selectOpenData.do?sno=11925%26publDataTpCd=%26searchCnd=%26searchWrd=%EC%A0%84%EA%B5%AD%2626pageIndex=1
	Total number of high‐cost medical equipment	Download from the Public Data Portal	Health Insurance Review & Assessment Service (HIRA)_ Detailed information service for hospital 05 Medical equipment information	2021	https://www.data.go.kr/data/15051055/fileData.do

Abbreviation: PXA, patient experience assessment.

### 2.3. Measures

#### 2.3.1. Dependent Variable

The dependent variable was the PXA, which measures patient experiences across six domains (nursing care service [four items], medical care service [four items], medication and treatment process [five items], hospital environment [two items], patient rights protection [four items], and overall evaluation) at the hospital level [16]. The individual items were assessed using a four‐point Likert scale, except for item 13 in the medication and treatment process domain, which used a two‐point scale (yes or no) [16]. The overall evaluation domain comprised two items measuring the comprehensive assessment of the hospitalization experience and willingness to recommend the hospital to others, assessed on an 11‐point scale [16]. The PXA scores, ranging from 0 to 100 points, were derived from individual items transformed using a linear scaling method. This process ensured that the scores for each item were consistently reported across all domains, with each domain represented by a score of 100 points [16].

### 2.4. Main Independent Variable

The independent variable was the percentage of patients receiving CNCS. This percentage represents the percentage of beds dedicated to CNCS out of the total number of beds in a hospital and was treated as a continuous variable ranging from 0 to 100. The proportion of CNCS beds was calculated using data provided by the Public Data Portal [17].

### 2.5. Associated Staffing Variable

NSG was an associated staffing variable. In South Korea, NSGs are determined based on the bed‐to‐nurse ratio at the hospital level (number of beds per nurse) and are categorized into seven levels from Grade 1 (*best*) to Grade 7 (*worst*) [22]. In this study, a binary variable was constructed by categorizing the bed‐to‐nurse ratio into those equal to or below 2.5:1 (tertiary hospitals: Grades 1 and 2; general hospitals: Grade 1) and those above 2.5:1 (tertiary hospitals: Grade 3; general hospitals: Grade 2 and above). Consequently, despite the differences in hospital type, NSGs were uniformly aligned at a ratio of 2.5:1 or less. This classification was used to standardize nurse staffing levels across hospital types and examine their association with PXAs.

### 2.6. Control Variables

This study controlled for the hospital characteristics known to influence the PXA. The control variables included hospital type, hospital ownership, location, hospital size, the percentage of beds in advanced hospital rooms, the percentage of medical specialists, and the total number of high‐cost medical equipment items. Hospital types were categorized into tertiary and general hospitals. Hospital ownership was classified as public/governmental or private. Hospital locations were categorized into the Seoul metropolitan area (Seoul, Incheon, and Gyeonggi) and other areas. Hospital size was categorized based on the number of beds into four categories: less than 300 beds, 301–500 beds, 501–700 beds, and 701 beds or more. Beds in advanced hospital rooms were defined as rooms accommodating three or fewer patients, compared with standard rooms with four or more beds. The percentage of beds in advanced hospital rooms refers to the proportion of beds in such rooms out of the total number of hospital beds [23]. Similarly, the percentage of medical specialists indicates the proportion of specialized physicians relative to the total number of doctors at each hospital. The total number of high‐cost medical equipment items was controlled as a continuous variable, considering the possession of 12 types of specialized, high‐cost devices and diagnostic radiology equipment by each hospital (CT scanners, MRI machines, mammography units, PET scanners, Gamma Knives, Cyber Knives, proton therapy devices, extracorporeal shock wave lithotripsy machines, hemodialysis units, ultrasound imaging devices, bone density scanners, and cone beam CT scanners).

### 2.7. Data Analysis

Data analysis was conducted using SAS (Version 9.4; SAS, Cary, NC, United States). Statistical significance was set at *p* < 0.05. Descriptive statistics, including the mean, standard deviation, frequency, and percentage, were calculated for all variables. A multivariate regression analysis was used to estimate the association of CNCS beds and NSFs with PXAs. A logistic regression analysis was used to estimate the association between CNCSs and NSGs. Hospital characteristics influencing PXA were included as control variables to construct the analytical model. Dummy variables were created for categorical variables such as hospital type, hospital ownership, and location. Continuous variables, including the percentage of beds in advanced hospital rooms, the percentage of medical specialists, and the total number of high‐cost medical equipment items, were also included in the analytic model.

## 3. Results

### 3.1. Characteristics of the Study Sample

**FIGURE 1:**
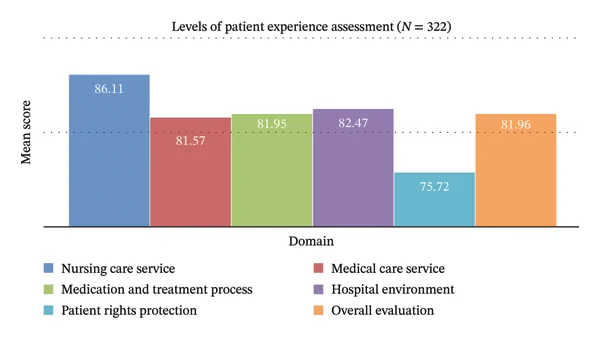
Levels of patient experience assessment.

Table 2 presents the descriptive statistics for the study variables used in the study. The average scores for each domain of the third PXA were as follows: nursing care service received an average score of 86.11, medical care service 81.57, medication and treatment process 81.95, hospital environment 82.47, patient rights protection 75.72, and overall evaluation 81.96 (Figure 1). Regarding NSGs, 67 hospitals (20.94%) were classified as Grade 1, corresponding to a bed‐to‐nurse ratio of less than 2.5:1 (less than 2.0:1 for tertiary hospitals). The average percentage of CNCS beds was 24.87%. Regarding hospital type, there were 47 tertiary hospitals (14.60%) and 275 general hospitals (85.40%). Public/government and private hospitals accounted for 47 (14.60%) and 275 (85.40%), respectively. Geographically, 118 hospitals (36.65%) were located in the Seoul metropolitan area. The average number of beds was 450.36, and the percentage of beds in advanced hospital rooms was 5.38%.

**TABLE 2:** Characteristics of the study sample.

Variables	Variable description	Third patient experience assessment, 2021	
		*N* (%)	Mean (SD)
*Patient experience assessment*		*N* = 322	
1. Nursing care service	1. Courtesy and respect		86.11 (3.36)
	2. Attentive listening		
	3. Explanation of hospital life		
	4. Commitment to handling requests		

2. Medical care service	5. Courtesy and respect		81.57 (3.31)
	6. Attentive listening		
	7. Opportunities to communicate with doctor information about doctor’s hospital rounds		
	8. Opportunities to communicate with doctor information about doctor’s hospital rounds		

3. Medication and treatment process	9. Explanation about medication/test/treatment		81.95 (3.46)
	10. Explanation about possible side effects		
	11. Efforts to control pain		
	12. Emotional support		
	13. Information about postdischarge care		

4. Hospital environment	14. Overall cleanliness		82.47 (5.49)
	15. Overall safety		

5. Patient rights protection	16. Fair treatment		75.72 (3.83)
	17. Ease of filing complaints		
	18. Opportunities for shared decision‐making		
	19. Consideration for shame, such as body exposure		

6. Overall evaluation	20. Overall hospital experience		81.96 (4.92)
	21. Intention to recommend		

*Nurse staffing grades*		*N* = 320	
Grade S	Less than 1.5		
Grade A	Less than 2.0:1		
Grade 1	2.0:1 or higher to less than 2.5:1 (less than 2.0:1 for tertiary hospitals)	67 (20.94)	
Grade 2	2.5:1 or higher to less than 3.0:1 (2.0:1 or higher to less than 2.5:1 for tertiary hospitals)	60 (18.75)	
Grade 3	3.0:1 or higher to less than 3.5:1 (2.5:1 or higher to less than 3.0:1 for tertiary hospitals)	42 (13.13)	
Grade 4	3.5:1 or higher to less than 4.0:1 (3.0:1 or higher to less than 3.5:1 for tertiary hospitals)	27 (8.44)	
Grade 5	4.0:1 or higher to less than 4.5:1 (3.5:1 or higher to less than 4.0:1 for tertiary hospitals)	28 (8.75)	
Grade 6	4.5:1 or higher to less than 6.0:1 (4.0:1 or higher for tertiary hospitals)	39 (12.19)	
Grade 7	6.0:1 or higher	57 (17.81)	
Missing		2	

*Percentage of comprehensive nursing care service beds*	Comprehensive nursing care service beds/total number of beds	*N* = 321	24.87 (25.45)
0%		90 (28.04)	
Less than 25%		110 (34.27)	
25%–50%		65 (20.25)	
50%–100%		56 (17.45)	
Missing		1	

*Hospital type*		*N* = 322	
Tertiary hospital		47 (14.60)	
General hospital		275 (85.40)	

*Hospital ownership*		*N* = 322	
Public/government		47 (14.60)	
Private		275 (85.40)	

*Location*		*N* = 322	
Seoul metropolitan area		118 (36.65)	
Others		204 (63.35)	

*Hospital size*		*N* = 322	450.36 (335.62)
Less than 300		163 (50.62)	
301–500		63 (19.57)	
501–700		38 (11.80)	
More than 701		58 (18.01)	

Percentage of beds in advanced hospital rooms	Number of beds in advanced hospital rooms/total number of beds	*N* = 322	5.38 (3.97)

Percentage of medical specialists	Number of medical specialists/total number of doctors	*N* = 322	85.64 (15.50)

*Total number of high-cost medical equipment*		*N* = 321	69.55 (52.92)
Missing		1	

Abbreviation: SD, standard deviation.

### 3.2. Association of the Percentage of CNCS Beds and NSGs With PXA: The Six Domains

Multiple linear regression was used to examine the association of the percentage of CNCS beds and NSGs with PXA across six domains: nursing care service (Models 1–6), medical care service (Models 7–12), medication and treatment process (Models 13–18), hospital environment (Models 19–24), patient rights protection (Models 25–30), and overall evaluation (Models 31–36).

Table 3 shows the association of the percentage of CNCS beds and NSGs with the nursing care service domain (Models 1–6). The adjusted R‐square values for these models ranged from 0.128 to 0.287, indicating varying levels of explanatory power. Model 1 revealed that hospitals with less than 25% CNCS beds, 25%–50%, and 50%–100% had better PXA scores than those without CNCS beds. Model 2, including the control variables, showed consistent results. Model 3 focused on NSGs, showing that bed‐to‐nurse ratios of 2.5:1 or less improved patient experience. In Model 4, which included the control variables, NSGs of 2.5:1 or less remained significant.

**TABLE 3:** Association of the percentage of comprehensive nursing care service beds and nurse staffing grades with patient experience assessment—nursing care service.

Variables	Model 1		Model 2		Model 3		Model 4		Model 5		Model 6	
	*β* (SE)	*p*	*β* (SE)	*p*	*β* (SE)	*p*	*β* (SE)	*p*	*β* (SE)	*p*	*β* (SE)	*p*
Intercept	84.236 (0.331)	< 0.001	86.240 (1.791)	< 0.001	85.293 (0.198)	< 0.001	87.457 (1.722)	< 0.001	84.143 (0.317)	< 0.001	86.214 (1.787)	< 0.001

*Percentage of comprehensive nursing care service beds*												
0% (ref)												
Less than 25%	3.074 (0.446)	< 0.001	1.432 (0.460)	0.002					2.142 (0.449)	< 0.001	1.442 (0.460)	0.002
25%–50%	2.373 (0.511)	< 0.001	1.246 (0.499)	0.013					1.459 (0.506)	0.004	1.024 (0.509)	0.045
50%–100%	1.960 (0.535)	< 0.001	1.475 (0.508)	0.004					1.585 (0.511)	0.002	1.386 (0.509)	0.007

*Nurse staffing grades (beds to nurse ratio)*												
More than 2.5:1 (ref)												
2.5:1 or less					3.253 (0.393)	< 0.001	1.194 (0.592)	0.045	2.620 (0.409)	< 0.001	1.149 (0.606)	0.059

*Hospital type*												
General hospital (ref)												
Tertiary hospital			−0.595 (0.868)	0.493			−1.121 (0.886)	0.207			−1.106 (0.906)	0.223

*Hospital ownership*												
Private (ref)												
Public/government			0.984 (0.469)	0.037			0.909 (0.475)	0.057			0.879 (0.471)	0.063

*Location*												
Others (ref)												
Seoul metropolitan area			0.441 (0.366)	0.228			0.442 (0.361)	0.223			0.358 (0.367)	0.33

*Hospital size*												
Less than 300 (ref)												
301–500			0.363 (0.469)	0.44			0.495 (0.468)	0.291			0.303 (0.469)	0.519
501–700			1.342 (0.682)	0.05			1.300 (0.690)	0.061			1.063 (0.694)	0.127
More than 701			2.482 (0.907)	0.007			2.278 (0.931)	0.015			2.059 (0.930)	0.028

Percentage of beds in advanced hospital rooms			0.175 (0.042)	< 0.001			0.177 (0.043)	< 0.001			0.171 (0.042)	< 0.001

Percentage of medical specialists			−0.035 (0.018)	0.055			−0.039 (0.018)	0.027			−0.033 (0.018)	0.066

Total number of high‐cost medical equipment			−0.000 (0.005)	0.999			−0.0001 (0.005)	0.977			−0.001 (0.005)	0.854

Adj. *R* ^2^	0.128		0.282		0.175		0.267		0.225		0.287	

*F* test	16.59		11.46		68.48		12.62		24.06		10.86	

*p*	< 0.001		< 0.001		< 0.001		< 0.001		< 0.001		< 0.001	

*N*	321		320		320		320		319		319	

*Note:* ref, reference group.

Abbreviation: SE, standard errors.

Model 5 demonstrated that the association of CNCS beds and NSGs with nursing care services remained significant. Hospitals with CNCS beds and a bed‐to‐nurse ratio of 2.5:1 or less showed better PXA scores. Model 6 included both the percentage of CNCS beds and NSGs, along with the control variables. All CNCS bed categories remained significant. Additionally, a hospital size of more than 701 beds and the percentage of beds in advanced hospital rooms were significant predictors. However, NSGs (bed‐to‐nurse ratio of 2.5:1 or less) were not a significant predictor in this model.

Table 4 shows the results for the medical care service domain (Models 7–12). Model 7 indicated that hospitals with CNCS beds had better PXA scores than those without them. In Model 8, the control variables, such as hospital size (more than 701 beds) and the percentage of beds in advanced hospital rooms, were also significant predictors, alongside the percentage of CNCS beds. Model 9 showed that NSGs of 2.5:1 or less improved PXA scores. Model 10, which included the control variables, supported the association of nurse staffing and hospital size as significant predictors. Model 11 revealed a significant combined effect of CNCS beds and NSGs. However, in Model 12, neither CNCS beds nor NSGs were significant when the control variables were included, whereas only the percentage of beds in advanced hospital rooms remained a significant predictor.

**TABLE 4:** Association of the percentage of comprehensive nursing care service beds and nurse staffing grades with patient experience assessment—medical care service.

Variables	Model 7		Model 8		Model 9		Model 10		Model 11		Model 12	
	*β* (SE)	*p*	*β* (SE)	*p*	*β* (SE)	*p*	*β* (SE)	*p*	*β* (SE)	*p*	*β* (SE)	*p*
Intercept	80.429 (0.341)	< 0.001	82.601 (1.937)	< 0.001	80.993 (0.205)	< 0.001	83.237 (1.837)	< 0.001	80.362 (0.336)	< 0.001	82.587 (1.931)	< 0.001

*Percentage of comprehensive nursing care service beds*												
0% (ref)												
Less than 25%	1.821 (0.459)	< 0.001	0.589 (0.498)	0.238					1.130 (0.476)	0.018	0.593 (0.497)	0.234
25%–50%	1.523 (0.526)	0.004	0.787 (0.539)	0.145					0.844 (0.536)	0.117	0.533 (0.551)	0.334
50%–100%	1.252 (0.550)	0.024	1.075 (0.550)	0.052					0.973 (0.541)	0.073	0.969 (0.550)	0.079

*Nurse staffing grades (beds to nurse ratio)*												
More than 2.5:1 (ref)												
2.5:1 or less					2.290 (0.407)	< 0.001	1.352 (0.632)	0.033	1.941 (0.433)	< 0.001	1.273 (0.655)	0.053

*Hospital type*												
General hospital (ref)												
Tertiary hospital			−0.451 (0.939)	0.631			−1.125 (0.946)	0.235			−1.019 (0.979)	0.299

*Hospital ownership*												
Private (ref)												
Public/government			0.444 (0.508)	0.382			0.308 (0.506)	0.543			0.327 (0.509)	0.521

*Location*												
Others (ref)												
Seoul metropolitan area			−0.401 (0.396)	0.312			−0.419 (0.385)	0.278			−0.495 (0.397)	0.213

*Hospital size*												
Less than 300 (ref)												
301–500			0.713 (0.508)	0.161			0.765 (0.500)	0.127			0.642 (0.507)	0.206
501–700			1.751 (0.737)	0.018			1.507 (0.736)	0.041			1.438 (0.750)	0.056
More than 701			2.390 (0.981)	0.015			1.976 (0.993)	0.048			1.918 (1.005)	0.057

Percentage of beds in advanced hospital rooms			0.149 (0.046)	0.001			0.149 (0.046)	0.001			0.146 (0.046)	0.002

Percentage of medical specialists			−0.031 (0.019)	0.112			−0.033 (0.019)	0.082			−0.030 (0.019)	0.129

Total number of high‐cost medical equipment			−0.005 (0.006)	0.389			−0.006 (0.006)	0.319			−0.006 (0.006)	0.294

Adj. *R* ^2^	0.042		0.129		0.088		0.139		0.096		0.137	

*F* test	5.69		4.95		31.69		6.15		9.46		4.87	

*p*	< 0.001		< 0.001		< 0.001		< 0.001		< 0.001		< 0.001	

*N*	321		320		320		320		319		319	

*Note:* ref, reference group.

Abbreviation: SE, standard errors.

Table 5 presents the results for the medication and treatment process domain (Models 13–18). Model 13 showed that hospitals with CNCS beds had better PXA scores. However, in Model 14, after adjusting for the control variables, the association was not significant for hospitals with less than 25% CNCS beds, while significant effects remained only for those with 25% or higher proportions. Model 15 indicated that nurse staffing ratios of 2.5:1 or less improved PXA scores, with similar results in Model 16 after adding control variables. Models 17 and 18 showed that CNCS beds and NSGs were positively associated with PXA scores. However, in Model 18, the effect of CNCS beds was significant only in hospitals with CNCS bed proportions above 50%. Additionally, public/government hospitals, larger hospital sizes, a higher percentage of beds in advanced hospital rooms, and a lower percentage of medical specialists were significant predictors.

**TABLE 5:** Association of the percentage of comprehensive nursing care service beds and nurse staffing grades with patient experience assessment—medication and treatment process.

Variables	Model 13		Model 14		Model 15		Model 16		Model 17		Model 18	
	*β* (SE)	*p*	*β* (SE)	*p*	*β* (SE)	*p*	*β* (SE)	*p*	*β* (SE)	*p*	*β* (SE)	*p*
Intercept	80.270 (0.348)	< 0.001	82.852 (1.878)	< 0.001	81.129 (0.204)	< 0.001	83.787 (1.789)	< 0.001	80.172 (0.332)	< 0.001	82.830 (1.871)	< 0.001

*Percentage of comprehensive nursing care service beds*												
0% (ref)												
Less than 25%	2.638 (0.469)	< 0.001	0.821 (0.483)	0.090					1.642 (0.470)	< 0.001	0.828 (0.482)	0.087
25%–50%	2.272 (0.537)	< 0.001	1.144 (0.523)	0.029					1.293 (0.530)	0.015	0.889 (0.533)	0.097
50%–100%	1.860 (0.562)	0.001	1.446 (0.533)	0.007					1.458 (0.535)	0.007	1.342 (0.533)	0.012

*Nurse staffing grades (beds to nurse ratio)*												
More than 2.5:1 (ref)												
2.5:1 or less					3.292 (0.406)	< 0.001	1.446 (0.615)	0.019	2.798 (0.428)	< 0.001	1.296 (0.635)	0.042

*Hospital type*												
General hospital (ref)												
Tertiary hospital			−0.064 (0.910)	0.944			−0.833 (0.921)	0.366			−0.641 (0.948)	0.5

*Hospital ownership*												
Private (ref)												
Public/government			1.127 (0.492)	0.023			0.982 (0.493)	0.047			1.008 (0.493)	0.042

*Location*			0.012 (0.383)	0.976			0.066 (0.375)	0.86			−0.084 (0.384)	0.828
Others (ref)												
Seoul metropolitan area												

*Hospital size*												
Less than 300 (ref)												
301–500			0.857 (0.492)	0.083			0.920 (0.487)	0.06			0.787 (0.491)	0.11
501–700			1.776 (0.715)	0.014			1.524 (0.717)	0.034			1.460 (0.727)	0.046
More than 701			2.554 (0.951)	0.008			2.113 (0.967)	0.03			2.075 (0.974)	0.034

Percentage of beds in advanced hospital rooms			0.171 (0.044)	< 0.001			0.171 (0.044)	< 0.001			0.168 (0.044)	< 0.001

Percentage of medical specialists			−0.040 (0.019)	0.036			−0.043 (0.018)	0.02			−0.038 (0.019)	0.043

Total number of high‐cost medical equipment			−0.003 (0.006)	0.656			−0.003 (0.006)	0.579			−0.004 (0.006)	0.52

Adj. *R* ^2^	0.091		0.255		0.169		0.253		0.196		0.261	

*F* test	11.61		10.09		65.81		11.78		20.37		9.66	

*p*	< 0.001		< 0.001		< 0.001		< 0.001		< 0.001		< .001	

*N*	321		320		320		320		319		319	

*Note:* ref, reference group.

Abbreviation: SE, standard errors.

Table 6 shows the results for the hospital environment domain (Models 19–24). Hospitals with CNCS beds had better PXA scores across all models. Nurse staffing ratios of 2.5:1 or less also improved PXA scores in both Models 21 and 22. The combined effect of CNCS beds and nurse staffing ratios remained significant in Models 23 and 24. Additionally, larger hospital sizes and the percentage of beds in advanced hospital rooms were significant predictors of better PXA scores.

**TABLE 6:** Association of the percentage of comprehensive nursing care service beds and nurse staffing grades with patient experience assessment—hospital environment.

Variables	Model 19		Model 20		Model 21		Model 22		Model 23		Model 24	
	*β* (SE)	*p*	*β* (SE)	*p*	*β* (SE)	*p*	*β* (SE)	*p*	*β* (SE)	*p*	*β* (SE)	*p*
Intercept	78.933 (0.532)	< 0.001	76.185 (2.957)	< 0.001	81.213 (0.326)	< 0.001	79.094 (2.897)	< 0.001	78.835 (0.512)	< 0.001	76.057 (2.942)	< 0.001

*Percentage of comprehensive nursing care service beds*												
0% (ref)												
Less than 25%	5.388 (0.717)	< 0.001	3.080 (0.760)	< 0.001					3.926 (0.726)	< 0.001	3.136 (0.758)	< 0.001
25%–50%	4.399 (0.822)	< 0.001	3.093 (0.823)	< 0.001					2.963 (0.818)	< 0.001	2.669 (0.839)	0.002
50%–100%	4.600 (0.859)	< 0.001	3.831 (0.839)	< 0.0001					3.985 (0.826)	< 0.001	3.678 (0.838)	< 0.001

*Nurse staffing grades (beds to nurse ratio)*												
More than 2.5:1 (ref)												
2.5:1 or less					5.102 (0.647)	< 0.001	2.652 (0.996)	0.008	3.989 (0.661)	< 0.001	2.382 (0.998)	0.018

*Hospital type*												
General hospital (ref)												
Tertiary hospital			−0.382 (1.433)	0.79			−1.716 (1.491)	0.251			−1.435 (1.491)	0.337

*Hospital ownership*												
Private (ref)												
Public/government			2.184 (0.775)	0.005			1.984 (0.798)	0.014			1.971 (0.775)	0.012

*Location*												
Others (ref)												
Seoul metropolitan area			0.737 (0.604)	0.223			0.898 (0.608)	0.14			0.574 (0.604)	0.343

*Hospital size*												
Less than 300 (ref)												
301–500			1.632 (0.775)	0.036			1.952 (0.788)	0.014			1.523 (0.772)	0.05
501–700			2.404 (1.125)	0.034			2.255 (1.160)	0.053			1.845 (1.143)	0.108
More than 701			4.533 (1.497)	0.003			4.018 (1.566)	0.011			3.670 (1.531)	0.017

Percentage of beds in advanced hospital rooms			0.183 (0.070)	0.009			0.183 (0.072)	0.011			0.173 (0.070)	0.014

Percentage of medical specialists			0.008 (0.030)	0.778			−0.003 (0.030)	0.918			0.012 (0.030)	0.686

Total number of high‐cost medical equipment			0.004 (0.009)	0.644			0.004 (0.009)	0.668			0.002 (0.009)	0.815

Adj. *R* ^2^	0.159		0.266		0.161		0.222		0.240		0.276	

*F* test	21.12		10.62		62.14		10.11		26.10		10.31	

*p*	< 0.001		< 0.001		< 0.001		< 0.001		< 0.001		< 0.001	

*N*	321		320		320		320		319		319	

*Note:* ref, reference group.

Abbreviation: SE, standard errors.

Table 7 presents the results for the protection of patient rights domain (Models 25–30). Overall, no consistent significant differences in PXA scores were observed across most CNCS bed categories or nurse staffing levels. In Model 30, only hospitals with 50%–100% CNCS beds showed a significant positive association with PXA scores. Additional significant factors included public/government hospitals, larger hospital sizes, and a higher percentage of beds in advanced hospital rooms.

**TABLE 7:** Association of the percentage of comprehensive nursing care service beds and nurse staffing grades with patient experience assessment—patient rights protection.

Variables	Model 25		Model 26		Model 27		Model 28		Model 29		Model 30		
	*β* (SE)	*p*	*β* (SE)	*p*	*β* (SE)	*p*	*β* (SE)	*p*	*β* (SE)	*p*	*β* (SE)	*p*	
Intercept	74.039 (0.388)	< 0.001	77.434 (2.122)	< 0.001	74.944 (0.233)	< 0.001	78.433 (2.027)	< 0.001	73.971 (0.378)	< 0.001	77.427 (2.125)	< 0.001	

*Percentage of comprehensive nursing care service beds*													
0% (ref)													
Less than 25%	2.723 (0.523)	< 0.001	0.886 (0.546)	0.105					1.786 (0.536)	0.001	0.888 (0.548)	0.106	
25%–50%	2.251 (0.599)	< 0.001	1.018 (0.591)	0.086					1.330 (0.605)	0.029	0.857 (0.606)	0.158	
50%–100%	1.749 (0.627)	0.006	1.325 (0.602)	0.029					1.358 (0.610)	0.027	1.258 (0.605)	0.039	

*Nurse staffing grades (beds to nurse ratio)*									2.570 (0.488)	< 0.001			
More than 2.5:1 (ref)													
2.5:1 or less					3.124 (0.462)	< 0.001	0.933 (0.697)	0.181	2.570 (0.488)	< 0.001	0.806 (0.721)	0.264	

*Hospital type*													
General hospital (ref)													
Tertiary hospital			−0.402 (1.028)	0.696			−0.924 (1.043)	0.376			−0.761 (1.077)	0.48	

*Hospital ownership*													
Private (ref)													
Public/government			1.272 (0.556)	0.023			1.187 (0.559)	0.034			1.198 (0.560)	0.033	

*Location*													
Others (ref)													
Seoul metropolitan area			0.110 (0.433)	0.8			0.150 (0.425)	0.724			0.050 (0.437)	0.909	

*Hospital size*													
Less than 300 (ref)													
301–500			0.617 (0.556)	0.268			0.719 (0.551)	0.193			0.572 (0.558)	0.306	
501–700			1.711 (0.807)	0.035			1.606 (0.812)	0.049			1.513 (0.826)	0.068	
More than 701			2.791 (1.074)	0.01			2.566 (1.096)	0.02			2.492 (1.106)	0.025	

Percentage of beds in advanced hospital rooms			0.219 (0.050)	< 0.001			0.222 (0.050)	< 0.001			0.217 (0.050)	< 0.001	

Percentage of medical specialists			−0.049 (0.021)	0.022			−0.054 (0.021)	0.011			−0.048 (0.021)	0.025	

Total number of high‐cost medical equipment			−0.006 (0.006)	0.351			−0.006 (0.006)	0.339			−0.007 (0.006)	0.3	

Adj. *R* ^2^	0.076		0.222		0.123		0.219		0.146		0.222		

*F* test	9.72		8.58		45.69		9.96		14.55		7.98		

*p*	< 0.001		< 0.001		< 0.001		< 0.001		< 0.001		< 0.001		

*N*	321		320		320		320		319		319		

*Note:* ref, reference group.

Abbreviation: SE, standard errors.

Table 8 shows the results for the overall evaluation domain (Models 31–36). In Model 31, hospitals with less than 25%, 25%–50%, and 50%–100% CNCS beds had better PXA scores than those with no CNCS beds. Model 32, which included the control variables, showed that hospitals with CNCS beds had better PXA scores. The significant control variables included public/government hospitals, hospital size, and the percentage of beds in advanced hospital rooms. Model 33 found that nurse staffing ratios of 2.5:1 or less improved PXA scores. Meanwhile, Model 34, which included the control variables, showed consistent results. In Model 35, the combined association of CNCS beds and NSGs remained significant; hospitals with CNCS beds and a nurse staffing ratio of 2.5:1 or less had better PXA scores. In Model 36, the percentage of CNCS beds continued to be a significant predictor even when NSGs and the control variables were included. However, NSGs were not significant predictors in this model. Significant control variables included public/government hospitals, a hospital size of more than 701 beds, and the percentage of beds in advanced hospital rooms.

**TABLE 8:** Association of the percentage of comprehensive nursing care service beds and nurse staffing grades with patient experience assessment—overall evaluation.

Variables	Model 31		Model 32		Model 33		Model 34		Model 35		Model 36	
	*β* (SE)	*p*	*β* (SE)	*p*	*β* (SE)	*p*	*β* (SE)	*p*	*β* (SE)	*p*	*β* (SE)	*p*
Intercept	79.216 (0.486)	< 0.001	81.294 (2.588)	< 0.001	80.751 (0.289)	< 0.001	83.249 (2.502)	< 0.001	79.082 (0.463)	< 0.001	81.221 (2.586)	< 0.001

*Percentage of comprehensive nursing care service beds*												
0% (ref)												
Less than 25%	4.388 (0.655)	< 0.001	1.748 (0.665)	0.009					2.973 (0.656)	< 0.001	1.780 (0.666)	0.008
25%–50%	3.586 (0.750)	< 0.001	2.078 (0.720)	0.004					2.196 (0.739)	0.003	1.803 (0.737)	0.015
50%–100%	3.045 (0.784)	< 0.001	2.697 (0.735)	< 0.001					2.472 (0.746)	0.001	2.595 (0.736)	< 0.001

*Nurse staffing grades (beds to nurse ratio)*												
More than 2.5:1 (ref)												
2.5:1 or less					4.855 (0.574)	< 0.001	1.805 (0.860)	0.037	3.963 (0.597)	< 0.001	1.525 (0.877)	0.083

*Hospital type*												
General hospital (ref)												
Tertiary hospital			−0.083 (1.254)	0.947			−1.112 (1.288)	0.389			−0.758 (1.311)	0.564

*Hospital ownership*												
Private (ref)												
Public/government			2.766 (0.678)	< 0.001			2.599 (0.690)	< 0.001			2.629 (0.682)	< 0.001

*Location*			−0.370 (0.528)	0.485			−0.229 (0.525)	0.663			−0.475 (0.531)	0.372
Others (ref)												
Seoul metropolitan area												

*Hospital size*												
Less than 300 (ref)												
301–500			0.558 (0.678)	0.412			0.764 (0.680)	0.262			0.486 (0.679)	0.474
501–700			2.135 (0.985)	0.031			1.940 (1.002)	0.054			1.774 (1.005)	0.078
More than 701			3.257 (1.310)	0.013			2.824 (1.352)	0.038			2.703 (1.346)	0.046

Percentage of beds in advanced hospital rooms			0.200 (0.061)	0.001			0.201 (0.062)	0.001			0.194 (0.061)	0.002

Percentage of medical specialists			−0.040 (0.026)	0.13			−0.048 (0.026)	0.063			−0.037 (0.026)	0.152

Total number of high‐cost medical equipment			0.004 (0.008)	0.586			0.004 (0.008)	0.616			0.003 (0.008)	0.707

Adj. *R* ^2^	0.124		0.3		0.181		0.280		0.228		0.304	

*F* test	16.11		12.39		71.56		13.41		24.44		11.68	

*p*	< 0.001		< 0.001		< 0.001		< 0.001		< 0.001		< 0.001	

*N*	321		320		320		320		319		319	

*Note:* ref, reference group.

Abbreviation: SE, standard errors.

### 3.3. Association of the Percentage of CNCS Beds With NSGs

Table 9 presents the results of a multivariate logistic regression analysis conducted on 319 hospitals, examining the association between the percentage of CNCS beds and NSG while controlling for several hospital characteristics. The results revealed that the percentage of CNCS beds is associated with better NSGs. Specifically, hospitals with less than 50% CNCS beds had 6.956 times higher odds of achieving better NSGs than hospitals with no CNCS beds (odds ratio [OR] = 6.956, 95% confidence interval [CI]: 1.076–44.975, *p* = 0.042). However, hospitals with more than 50% CNCS beds and those with no CNCS beds exhibited no statistically significant differences (OR = 6.403, 95% CI: 0.833–49.235, *p* = 0.074).

**TABLE 9:** Association of the percentage of comprehensive nursing care service beds with nurse staffing grades (*N* = 319).

Variables	Model 37 nurse staffing grades (bed‐to‐nurse ratio)		
	OR	95% CI	*p*
*Percentage of comprehensive nursing care service beds*			
0% (ref)	1		
Less than 50%	6.956	1.076–44.975	0.042
More than 50%	6.403	0.833–49.235	0.074

*Hospital type*			
General hospital (ref)	1		
Tertiary hospital	27.755	2.480–310.590	0.007

*Hospital ownership*			
Private (ref)	1		
Public/government	3.282	1.010–10.666	0.048

*Location*			
Others (ref)	1		
Seoul metropolitan area	2.864	1.136–7.220	0.026

*Hospital size*			
Less than 300 (ref)	1		
301–500	0.928	0.233–3.696	0.915
501–700	1.339	0.241–7.430	0.739
More than 701	1.381	0.147–12.987	0.778

Percentage of beds in advanced hospital rooms	1.010	0.915–1.114	0.849

Percentage of medical specialists	0.995	0.959–1.032	0.779

Total number of high‐cost medical equipment	1.037	1.013–1.061	0.003

*Note:* ref, reference group.

Abbreviations: CI, confidence interval; OR, odds ratio.

Similarly, tertiary hospitals had higher odds of achieving better staffing grades than general hospitals, and public/government hospitals also showed higher odds compared to private hospitals. Additionally, hospitals located in the Seoul metropolitan area had higher odds of achieving better staffing grades than those in other regions, whereas the total number of high‐cost medical equipment items was a significant factor positively contributing to better staffing levels. Thus, CNCS beds may support better staffing levels, with tertiary and public hospitals generally exhibiting more favorable nurse staffing levels.

## 4. Discussion

This study highlighted the CNCS’ contribution to patient satisfaction in South Korean hospitals, as it helps alleviate caregiving burdens in terms of both costs and the logistical challenges faced by patients’ families.

Nursing care services emerged as a significant factor in enhancing patient experience. That is consistent with Hwang and Park [11], who found that, compared to family caregiving, comprehensive nursing care significantly increased inpatient satisfaction. Their study also highlighted that nurses’ positive attitudes and behaviors were strongly associated with higher levels of patient satisfaction. The consistent positive association of the CNCS with patient experience, as demonstrated in the study, reinforces the importance of high‐quality nursing care in contributing to patient outcomes.

However, Choi et al. [7] found that patient experience scores in the nursing domain were not significantly related to the proportion of CNCS beds. Instead, they identified a significantly positive relationship between the implementation level of these beds and patient experience in the hospital environment domain, along with a marginally significant relationship in the overall evaluation. Similarly, the present study found that the percentage of CNCS beds may significantly contribute to the hospital environment and overall evaluation.

Additionally, this study found that NSGs were associated with patient experiences. The CNCS policy is intended to improve nurse staffing levels, which may enhance patient outcomes. We found that better staffing levels were associated with better patient experience in some domain models, which may support a potential pathway through which CNCS implementation may influence patient outcomes. However, this relationship was not consistent across all domains or fully adjusted models, indicating that the contribution of nurse staffing is likely partial.

Hospitals with NSGs or lower generally showed better patient experience scores in some models, supporting the importance of adequate staffing. However, these associations were not consistent across all domains and models. Similarly, Song and Do [12] identified a positive relationship between patient experience in the nursing domain and NSGs, even when controlling for hospital characteristics and individual patient factors. For instance, they found that the probability of patients selecting the top response of “Always” to the question “How often did nurses treat you with courtesy and respect?” was 70.3% in nontertiary general hospitals with the highest NSG, compared with 63.1% in hospitals with the lowest NSG. Similar associations were observed in other patient experience domains, which were likely influenced by nurse staffing levels, although these associations were generally weaker and less consistent than those found in the nursing domain.

Furthermore, the findings varied across patient experience domains. The association between CNCS beds and patient experience was most consistent in the hospital environment domain, where significant effects were observed across models. In the nursing care service and overall evaluation domains, CNCS beds were significantly associated with patient experience, whereas NSGs were not significant in the fully adjusted models.

In contrast, no significant associations were observed in the medical care service domain, suggesting that the CNCS and nurse staffing may have limited influence on physician‐related aspects of care. For the medication and treatment process domain, CNCS beds were significantly associated with patient experience only at higher levels (50%–100%), while NSGs remained significant. For the patient rights protection domain, only hospitals with 50%–100% CNCS beds showed a significant association.

These findings highlight the importance of adequate nurse staffing in the design and implementation of CNCS policies. In this study, NSGs were significantly associated with patient experience in the medication and treatment process and hospital environment domains, suggesting that staffing may play a role in specific aspects of patient care. In the United States, California implements this type of nurse staffing policy for all types of nursing units, while Massachusetts implements it only for intensive care units [24]. Thus, the global implications of this study suggest that service models such as the CNCS, coupled with adequate staffing, may inform international debates on safe staffing ratios and patient‐centered care delivery.

This study had several limitations. Because this study consisted of a secondary analysis using data from HIRA, the ability to control for all potential variables was limited. Additionally, the variability in the composition of nursing and nursing unit support staff across hospitals introduced significant differences in the operations, work environment, and quality of the CNCSs. The lack of detailed information on the adequacy and specific operational aspects of these services further limits the scope of the findings. Another limitation was the cross‐sectional design, which restricted causal inference from the study findings. Future research should incorporate these variables to provide a more nuanced understanding of their effects on patient outcomes. In particular, future studies should investigate the association between the CNCSs and clinical outcomes, such as infection rates, recovery times, and length of stay, and patient experience in specialized units such as surgical nursing units.

## 5. Conclusion

The CNCS if reinforced as a critical factor contributing to patient satisfaction. By reducing the burden of caregiving costs and providing consistent, high‐quality nursing care, these services improve the overall patient experience and contribute to more favorable patient outcomes. Future research should continue to explore the interplay among nurse staffing levels, the CNCS, and patient satisfaction to inform policies that can sustain and enhance the quality of healthcare delivery in South Korea.

### 5.1. Implications for Nursing Management

The findings have significant implications for nursing management and healthcare policy in South Korea. Hospitals with a higher proportion of CNCS beds—particularly those exceeding 50% of total beds—were associated with significantly better patient experience scores across the nursing care, medication and treatment, hospital environment, and overall evaluation domains. Policymakers should focus on providing adequate nurse staffing and expanding the CNCS to improve patient satisfaction and healthcare quality. In terms of workforce planning, nurse managers should also prioritize staffing allocation in units implementing the CNCS. Furthermore, from a monitoring and resource allocation perspective, the domain‐specific pattern of associations provides nurse managers with a targeted framework for patient experience monitoring and quality improvement. The hospital environment and nursing care service domains showed the most consistent relationship with CNCS implementation across all adjusted models, suggesting that they may serve as primary indicators for evaluating CNCS effectiveness. Overall, hospital administrators should prioritize resource allocation to ensure adequate staffing and organizational support, allowing hospitals to effectively meet patient needs.

## Author Contributions

Sung‐Heui Bae: conceptualization, methodology, supervision, writing–original draft preparation, writing–review and editing. Ye‐Eun Jin: formal analysis, data curation, writing–original draft preparation, writing–review and editing.

## Funding

This research was supported by the BK21 FOUR (Fostering Outstanding Universities for Research) funded by the Ministry of Education (MOE, Korea) and National Research Foundation of Korea (NRF‐5199990614253).

## Ethics Statement

This study did not involve the collection or analysis of human participant data. The Institutional Review Board of Ewha Womans University determined that formal ethical approval was not required, and IRB approval was waived for this study.

## Conflicts of Interest

The authors declare no conflicts of interest.

## Data Availability

The data that support the findings of this study are openly available from the Health Insurance Review & Assessment Service (HIRA) at https://www.hira.or.kr/ra/eval/getDiagEvlList.do?pgmid%3dHIRAA030004000100.
